# Evaluation of Exposure to Bisphenol A, Bisphenol F, and Phthalates in Patients with Phenylketonuria and Its Differences According to Dietary Status

**DOI:** 10.3390/nu16183213

**Published:** 2024-09-23

**Authors:** İzzet Erdal, Yılmaz Yıldız, Sıddıka Songül Yalçın, Anıl Yirün, Göksun Demirel, Pınar Erkekoğlu

**Affiliations:** 1 Clinic of Pediatric Metabolic Diseases, Etlik City Hospital, 06170 Ankara, Türkiye; 2 Division of Social Pediatrics, Department of Pediatrics, Hacettepe University İhsan Doğramacı Children’s Hospital, 06230 Ankara, Türkiye; siyalcin@hacettepe.edu.tr; 3 Division of Pediatric Metabolism, Department of Pediatrics, Hacettepe University İhsan Doğramacı Children’s Hospital, 06230 Ankara, Türkiye; yilmaz.yildiz@hacettepe.edu.tr; 4 Department of Pharmaceutical Toxicology, Faculty of Pharmacy, Çukurova University, 01330 Adana, Türkiye; anil.yirun@gmail.com (A.Y.); demirelgoksun3@gmail.com (G.D.); 5 Department of Pharmaceutical Toxicology, Faculty of Pharmacy, Hacettepe University, 01330 Ankara, Türkiye; erkekp@hacettepe.edu.tr; 6 Department of Vaccine Technology, Vaccine Institute, Hacettepe University, 06230 Ankara, Türkiye

**Keywords:** phenylketonuria, bisphenol, phthalate, environmental pollutant, endocrine-disrupting chemicals, plasticizer

## Abstract

Background: Phenylketonuria (PKU) is the most common amino acid metabolism disorder. Patients with blood phenylalanine (Phe) levels of ≥6 mg/dL require treatment, and the most definitive treatment is the Phe-restricted diet. Bisphenols and phthalates are widely used endocrine-disrupting chemicals (EDCs) found in personal care products, baby bottles, and food packaging. Methods: In this study, we evaluated the possible routes of exposure to these EDCs in patients diagnosed with PKU (n = 105, 2–6 years of age) and determined the relationship between the plasma levels of bisphenol A (BPA), bisphenol F (BPF), di-butyl phthalate (DBP), di-(2-ethylhexyl) phthalate (DEHP), mono-(2ethylhexyl) phthalate (MEHP), and dietary regimens. Participant characteristics and exposure routes were evaluated according to their dietary treatment status. Results: Thirty-four of these patients were on a Phe-restricted diet, while the remaining 71 had no dietary restrictions. DBP and DEHP levels were higher in those using plastic tablecloths (*p* = 0.049 and *p* = 0.04, respectively). In addition, plasma DBP levels were higher in those who used bottled water (*p* = 0.01). Being under 4 years of age, using plastic food containers, and using plastic shower curtains were characteristics associated with higher MEHP levels (*p* = 0.027, *p* = 0.019, and *p* = 0.014, respectively). After adjustment for baseline characteristics (Model 1), the odds of having a plasma BPA level in the upper tertile were 3.34 times higher in the free-diet group (95% CI = 1.09–10.25). When we additionally adjusted for plastic exposure (Model 2), the odds ratio was found to be 18.64 (95% CI = 2.09–166.42) for BPA. In the free-diet group, the probability of having plasma DEHP levels in the upper tertile was increased by a relative risk of 3.01 (*p* = 0.039, 95% CI = 1.06–8.60). Conclusion: Our results indicate that exposure to bisphenols and phthalates varies with dietary treatment. The difference in sources of exposure to EDCs between the diet and non-diet groups indicates that diet plays an important role in EDC exposure.

## 1. Introduction

Phenylketonuria (PKU) is the most common amino acid metabolism disorder, caused by mutations in the phenylalanine hydroxylase (PAH) gene [1]. Although its prevalence is approximately 1:10,000 worldwide, the frequency is approximately 1:4000 in Türkiye, where consanguineous marriages are common and PKU can be encountered by autosomal recessive inheritance [2,3]. High levels of phenylalanine (Phe) in the blood cause toxic effects, especially in the brain, and untreated patients may have symptoms such as low IQ, cognitive retardation, emotional and psychiatric problems, eczema, autism, seizures, and motor loss [1]. In many countries, thanks to newborn screening programs, patients are diagnosed during the neonatal period and treatment is started before the onset of symptoms [3]. In PKU patients, treatment needs are determined by blood Phe levels. In healthy individuals, blood Phe levels range from 0.8 to 1.99 mg/dL. Patients with blood Phe levels of ≥6 mg/dL require treatment, and the best known and most definitive treatment is the Phe-restricted diet. Sapropterin dihydrochloride, a synthetic form of tetrahydrobiopterin (BH4, cofactor of the PAH enzyme), is another treatment option, and BH4-responsive patients can continue their treatment without the need for a Phe-restricted diet [4,5,6,7]. The target of treatment is to keep Phe levels <6 mg/dL in children under 12 years of age and <10 mg/dL in those 12 years of age and older [6]. The amount of natural protein that can be taken by patients will vary depending on their level of Phe tolerance. The remainder of the protein needed for normal growth and development is provided by Phe free amino acid mixtures. These amino acid mixtures are offered in special packaging in powder or liquid form. Plastic packaging is usually used for liquid forms and metal packaging is used for powder forms, but in both cases there is a plastic-based coating inside the package/box [1].

Today, many environmental pollutants that result from human activities contaminate the water, soil, and air. These chemicals, directly or indirectly, can lead to acute or chronic health problems, and exposure to these chemicals is suggested to be responsible for approximately 9 million deaths each year [8]. These pollutants include plasticizers (phthalates, bisphenols, etc.), heavy metals, and many other substances (polychlorinated biphenyls, polucyclic aromatic hydrocarbons, etc.) [9]. Phthalates, which were first used as plasticizers in the 1930s, are now widely used in consumer and personal care products [10]. Low molecular weight phthalates are often added to personal care products to preserve odor while high molecular weight phthalates are used in polyvinyl chloride (PVC) to increase flexibility. Phthalates are found in a variety of products, including flooring, food packaging, and medical tubing. Phthalates do not covalently bind to plastic matrices and are easily separated from polymers. Humans can be exposed to these chemicals by the oral route (i.e., by food and water) [11]. Phthalates are well known for their endocrine-disrupting effects, and they are associated with metabolic syndrome, neurodevelopmental disorders, urogenital changes/malformations, alterations in embryogenesis, thyroid dysfunction, obesity, diabetes, cardiovascular disease, and autoimmune and inflammatory diseases [11,12,13,14,15,16,17,18,19,20,21,22]. Bisphenol A (BPA) is another of the widely exposed chemicals, with more than 2 million tons produced annually. It is used to harden polycarbonate plastics and epoxy resins. Polycarbonate plastics are used in baby bottles, food storage containers, water bottles and bottle caps, eyeglass lenses, CDs, DVDs, and electronic devices [23]. Studies have also shown that early BPA exposure has been linked to adverse neurodevelopmental effects, heart disease, diabetes, elevated liver enzymes, and impaired thyroid function [24,25,26,27]. BPA-containing baby bottles are banned in Europe for use by infants <3 years of age. Due to their consumption of more food according to body mass, immature detoxification mechanisms, and increased susceptibility to harmful effects of endocrine-disrupting chemicals (EDCs), children are more likely to face the toxic effects of bisphenols [28]. Türkiye has prohibited the manufacture of plastics containing BPA for use in infant and toddler feeding bottles [29]. Due to increasing awareness and restrictions against BPA, bisphenol F (BPF), one of the bisphenol analogues produced by manufacturers as an alternative, has become widely used today. It can be used in almost any application where the use of BPA is restricted. Most of the materials promoted as BPA-free contain other bisphenol analogues, primarily BPF [30]. For example, the use of BPA is banned in infant formula and the interior surface coating of its containers, and in the manufacture of infant and toddler feeding containers, but there is no regulation for other bisphenol analogs [30,31]. 

Oral exposure to EDCs is increasing every year due to the high production of these chemicals [11,32,33]. In this study, we evaluated the possible routes of exposure to phthalates and bisphenols in patients diagnosed with PKU and determined the relationship between the plasma levels of BPA, BPF, di-butyl phthalate (DBP), di-(2-ethylhexyl) phthalate (DEHP), mono-(2ethylhexyl) phthalate (MEHP), and dietary regimens. Our findings can provide valuable insights into the potential risks associated with the specialized dietary products used in PKU patients and may lead to future regulations to minimize the exposure to harmful chemicals in this susceptible population.

## 2. Materials and Methods

The study protocol was approved by the Hacettepe University Ethics Committee (GO 22/659). The study was conducted between July 2022 and January 2024 and included a total of 105 patients aged 2–6 years with diagnoses of PKU. The patients included in the study were diagnosed with PKU during the neonatal period through the newborn screening program and were being clinically followed at the center where the study was conducted. Written informed consent was obtained from the participating children and their families before their enrollment to the study. The patients were subject to physical examination: the anthropometric measurements (height and weight) of all participants were taken and age-related body mass index (BMI) z-scores (BAZ) were calculated. The data regarding the patients’ diseases, including the age at diagnosis, Phe levels, and the treatments they were receiving, were obtained from hospital records.

### 2.1. Sample Collection

All glassware was cleaned, kept in 10% nitric acid solution for 24 h, and then rinsed 4 times with distilled deionized water. Glass tubes were kept free of plasticizers by keeping them at 400 °C for 4 h. During the study, aluminum foil was used in order to prevent contact with the plastic material on the lids of all glass materials. Blood samples were collected in the morning upon admission to the hospital for routine outpatient clinic monitoring and then centrifuged at 3500 rpm for 10 min. The plasma samples obtained were transferred to −80 °C in deplasticized amber vials and stored until the analysis day. 

### 2.2. Questionnaire

The parents of the participants were questioned about their socio-demographic characteristics and were administered a questionnaire on exposure to environmental pollutants. These questions included patients’ week of birth, birth weight and type, breastfeeding status, birth order, total number of children, economic status, and sources of children’s exposure to plasticizers.

### 2.3. Measurement of Plasma Bisphenol Levels

All chemicals were purchased from Sigma-Aldrich (St. Louis, MI, USA). Glucuronidase/aryl sulfatase enzyme (from *Helix pomatia*) was from Roche Diagnostic GMBH (Lot No: 55244423, Ref: 10127698001, Mannheim, Germany). High-pressure liquid chromatography (HPLC) equipment was obtained from Agilent 1260 Quat Pump (Serial No: DEAB818844, Santa Clara, CA, USA).

The levels of BPA and BPF in plasma samples were determined with a method based on the extraction of bisphenols from plasma with ammonium acetate buffer and n-hexane: diethyl ether, followed by evaporation under nitrogen gas. The residue was dissolved in the mobile phase and quantified by high-performance liquid chromatography (HPLC) [34]. The limits of detections (LODs) were 3.05 ng/mL for BPA and 3.24 ng/mL for BPF. The limits of quantification (LOQs) were 13.1 ng/mL for BPA and 7.0 ng/mL for BPF. After extraction, the samples stored at −20 °C were dissolved in 300 μL 60% acetonitrile (ACN). Standards and samples were injected to HPLC as 100 μL. The mobile phase comprised acetonitrile and 2.5% (*v*/*v*, in water) tetrahydrofuran. Its flow rate was 0.4 mL/min. Gradient elution was applied as 60:40 to 5:95. Retention times for BPA and BPF were 18.4–18.7 min and 10.4–11.2 min, respectively. Recovery studies were performed on blank samples of plasma spiked with levels of 10 μg/mL of BPA and BPF. Within-day precision was 4.87 ± 3.02% coefficient of variation (CV) for BPA and 5.65 ± 1.20% CV for BPF. Between-run precision was 9.15 ± 2.58% CV for BPA and 9.84 ± 3.14% CV for BPF. As a result of 10 different analyses in recovery studies, recovery BPA was determined to be 81.64 ± 2.48% and 86.9 ± 1.87% for BPF. 

### 2.4. Determination of Plasma Phthalate Levels

For the analyses of plasma DEHP, DBP, and MEHP levels, the method of Paris et al. (2003) was used, with some modifications [35]. Briefly, after spiking plasma (200 µL) with phthalates (1 ppm in the last volume), NAOH (1N, 400 µL), phosphoric acid (H_3_PO_4_, 50%, 100 µL), and ACN (800 µL) were added and mixed. The mixture was vortexed and centrifuged. Supernatant (600 µL) was taken into another tube and evaporated until dryness under nitrogen stream. Residues were stored at −20 °C.

On analysis date, residues were dissolved in 60% ACN (300 µL). Standards (0.2, 0.5, 1, 2, and 5 ppm for DEHP; 0.2, 0.5, 1, 2, and 5 ppm for DBP; 0.2, 0.5, 1, 2.5, and 5 ppm for MEHP) and samples (100 µL) were injected into HPLC (Agilent 1100 series, Santa Clara, CA, USA). The HPLC columns were Spherisorb C18 ODS2 columns (25 cm × 5 µm × 4.6 mm i.d.) (Waters, Milford, MA, USA), and ODS C18 precolumns (4 cm) (Waters, Milford, MA, USA). The mobile phase was 0.1% H_3_PO_4_ and ACN [pH 3.0, 80:20 (*v*/*v*)]. Flow rate was 1 mL/min. The retention times for DBP, DEHP, and MEHP were 4.1 min, 32.5 min, and 4.5 min, respectively. Due to the close retention times of DBP and MEHP, their analyses were performed separately. The plasma concentrations of DBP, DEHP, and MEHP were calculated from standards and peak areas were used for quantication. The LODs were 0.38 µg/mL for DBP, 0.09 µg/mL for DEHP, and 1.41 µg/mL for MEHP. The LOQs were 1.15 µg/mL for DBP, 0.27 µg/mL for DEHP, and 4.26 µg/mL for MEHP. Recovery studies were performed on blank samples of plasma spiked with levels of 9.1 µg/mL of DBP, 9.8 µg/mL of DEHP, and 10.1 µg/mL of MEHP. The within-day precisions were 0.71 ± 0.40% CV for DBP, 3.09 ± 1.29% CV for DEHP, and 3.27 ± 1.05% CV for MEHP. The between-run precisions were 1.06 ± 0.56% CV for DBP, 9.21 ± 1.19 CV for DEHP, and 7.92 ± 2.11% CV for MEHP.

### 2.5. Matrix Spiking

As the samples had bisphenol and/or phthalate levels lower than LOD, we used matrix spiking. For matrix spiking, we used phthalate or bisphenol spikes at known concentrations and used two proportions of a sample: matrix spike portion and test sample portion. In the “matrix spike” portion, we added a known amount of standard to increase the concentration by a known amount. When we tested the sample and then the matrix spike, the matrix spike resulted higher by the known amount added. The level of spiking was chosen according to the HPLC method used and was validated for at least 10 samples for each bisphenol/phthalate derivative. Although matrix spiking was used, some of the samples did not contain bisphenol and/or phthalate derivatives and the levels of bisphenol/phthalate derivatives found after analyses were not higher than the matrix spike levels. These samples were judged as non-detectable (nd), which means the level of the analyte in the sample is lower than LOD or is simply zero. The recovery rate (%R) was calculated by subtracting the level of matrix spike from the obtained result and multiplying the obtained result by 100. Briefly,

(%R) = (ng/mL spiked result − ng/mL spike) × 100.

In our study, all samples showed detectable bisphenol and/or phthalate levels with matrix spiking [36,37].

### 2.6. Statistical Analysis

Data analysis was performed using IBM SPSS Statistics for Windows, Version 23.0 (Chicago, IL, USA). The distribution of the data was evaluated through skewness, kurtosis, histograms, and the Kolmogorov–Smirnov test. Descriptive statistics were expressed as means with standard deviations (SD), medians with interquartile ranges (IQR), and percentages. For intergroup comparisons, analyses were conducted when each independent group included at least 10 cases (approximately 10% of the total sample size). The differences in plasticizer levels according to the categorical independent variable were analyzed using the Mann–Whitney U test for two subgroups and the Kruskal–Wallis ANOVA test for three or more subgroups. In cases where significant differences were found in variables having more than two groups, post-hoc subgroup analysis was conducted using Bonferroni correction. 

Chi-square tests were utilized to assess the association between diet status and plasticizer levels (upper tertile and others). Multiple logistic regression was used to investigate the association between being in the upper tertile of plasticizer levels and diet status (free diet versus PA-restricted diet), adjusting for age, gender (female vs. male), body mass index (BMI) for age z-score (BAZ), birth order, and total breastfeeding duration in Model 1. Model 2 was expanded by incorporating (e.g., baby bottles, shampoo, food containers, water heaters, frozen food, canned food, packaged food, canned beverages, PVC, shower curtains, tablecloths, cartridge printers, room sprays, recent carpet or furniture purchases, use of plastic during food preparation and/or consumption, water, and toys) alongside the variables controlled in Model 1. Adjusted odds ratios (AORs) and 95% confidence intervals (CI) were calculated. Significance was determined at *p* < 0.05.

## 3. Results

A total of 105 patients were included in the study, including 38 with hyperphenylalaninemia (HPA, those with blood Phe levels between 2 and 6 mg/dL without treatment at the time of diagnosis), 37 with BH4-responsive PKU, and 30 with PKU (those with a Phe-restricted diet). However, one patient with HPA did receive a Phe-restricted diet, as plasma Phe levels were above 6 mg/dL during follow-up. A total of 34 BH4-responsive PKU patients were treated with BH4 monotherapy, while 3 patients were also receiving Phe-restricted diet therapy. A total of 32.4% (n = 34) of the patients were on a Phe-restricted diet. The mean age of participants was 45.3 months, and 46.7% were male. The median age at diagnosis was 18 days, and the mean Phe level at diagnosis was 6.2 mg/dL. The mean of the patients’ last three plasma PA measurements was 3.06 mg/dL, and 91.4% (n = 96) were below the treatment goal of 6 mg/dL. The percentage of mothers and fathers with >8 years of education was lower (42% and 41% for mothers and fathers, respectively). A total of 50.5% of the participants indicated that their income was sufficient to cover their expenses (Table 1).

The median levels of plasticizers were 20.13 ng/mL for BPA, 6.06 ng/mL for BPF, 0.23 ng/mL for DBP, 0.48 ng/mL for DEHP, and 0.52 ng/mL for MEHP (Table 2).

### 3.1. Overall Study Group

There were no differences between BPA levels concerning participant characteristics. Plasma Phe levels were observed to be higher in the free-diet group, although there was no statistical significance. BPF levels were lower in those who used plastic shower curtains than in those who did not (*p* = 0.013). DBP and DEHP levels were higher in those using plastic tablecloths (*p* = 0.049 and *p* = 0.04, respectively). In addition, plasma DBP levels were higher in those who used bottled water (*p* = 0.01). Being <4 years of age, using plastic food containers, and using plastic shower curtains were characteristics associated with higher MEHP levels (*p* = 0.027, *p* = 0.019, and *p* = 0.014, respectively) (Table 3). 

After adjustment for baseline characteristics (Model 1), the odds of having a plasma BPA level in the upper tertile were 3.34 times higher in the free-diet group (95% CI = 1.09–10.25). When we additionally adjusted for plastic exposure (Model 2), the odds ratio (OR) was found to be 18.64 (95% CI = 2.09–166.42) for BPA. The probability of having plasma DEHP levels in the upper tertile was increased by 3.01 times (95% CI = 1.06–8.60) in Model 1 in the free-diet group. However, this increased risk was no longer significant when sources of plastic exposure were also included in the equation (Model 2) (Table 4).

### 3.2. PA-Restricted Diet Group

No significant associations were found between BPA, BPF, DBP, DEHP, and MEHP levels and any participant characteristics (Table S1).

### 3.3. Free-Diet Group

Bisphenol A levels were significantly higher in patients who used plastic food containers than in those who received no dietary treatment (29.88 ng/mL vs. 17.41 ng/mL, *p* = 0.041). BPF levels were also higher in those who did not use plastic shower curtains than in those who used them (*p* = 0.011 and *p* = 0.013, respectively). DBP levels were higher in those who used bottled water for cooking and/or drinking (*p* = 0.028 and *p* = 0.004, respectively). Total number of children (*p* = 0.017), paternal education level (*p* = 0.021), use of canned food (*p* = 0.044), and use of plastic shower curtains (*p* = 0.017) were found to be associated with higher plasma MEHP levels (Table S2). 

## 4. Discussion

This study is the first in the literature to evaluate the exposure of PKU patients to multiple environmental EDCs. Our results suggest that the exposure of patients with PKU to EDCs varies according to their treatment regimen. Patients on a free diet have a higher odds for exposure to plasticizers compared to those on a restricted diet. It can be suggested that different dietary treatment regimens can affect plasma EDC levels. The results are discussed under two sub-titles.

### 4.1. Bisphenols

The median plasma BPA levels of the participants in our study were found to be 20.13 ng/mL. In a study conducted in Türkiye, the mean serum BPA level in healthy individuals between the ages of 13 and 19 was reported to be 0.8 ng/mL, which is lower than the level observed in our study [38]. Kondolot et al. reported that the median plasma BPA level in 50 healthy children with a mean age of 5.6 years was 1.12 ng/mL [39]. In a study comparing cryptorchidism patients aged 1–4 years with healthy controls, median BPA and BPF levels were reported to be 5.54 ng/mL and 1.83 ng/mL in the healthy control group [40]. Plasma BPA levels were found to be higher in our study compared to studies conducted in both Türkiye and other countries. Since the study did not include a healthy control group, it is not possible to say with certainty whether or not the cause of this elevated level is related to the disease. However, no relationship between phenylalanine metabolism and BPA metabolism has been reported in the literature.

The primary analyses revealed no significant differences in plasma BPA levels according to any participant characteristics in the overall group (Table 3). However, on further analysis, we found that people who had a free diet were more likely to have BPA levels in the upper tertile (Table 4). These were patients who follow a free diet with no restrictions on the consumption of foods high in protein, especially meat and meat products, milk, eggs, and fish. At the same time, there is no need to restrict vegetable consumption. However, in the Phe-restricted diet group, they can hardly consume meat and meat products, fish, milk, eggs, etc., and while there is no restriction on the consumption of most vegetables, they can still consume some vegetables to a limited extent [1,6]. The BPA content of animal foods is responsible for 10–50% of human exposure to BPA [41]. Even unpackaged, freshly expressed milk has been shown to contain measurable levels of BPA, as demonstrated by Santonicola et al. (2019) [42]. There are studies that report similar or higher levels of BPA in vegetables as well as in high-protein animal foods [31,43,44,45]. Patients in the Phe-restricted diet group would not have been exposed to BPA from animal foods due to their restricted diet. Furthermore, if they consumed vegetables, this would have limited their exposure to BPA, as they consumed them in a limited quantity. For these reasons, it can be postulated that the Phe-restricted group had a reduced exposure to BPA.

When the exposure to environmental EDCs was evaluated according to the participant characteristics of the subgroups according to their dietary status, BPA was measured higher in those who used plastic food containers in the free-diet group (29.88 vs. 17.41, *p* = 0.041). BPA is one of the most widely produced plasticizer chemicals today and is used in the food chain and in containers used to store food. It leaches from these containers into food and is absorbed by the gastrointestinal system during consumption [46]. Patients receiving diet therapy are restricted in their diet to foods rich in natural protein, such as meat, milk, and eggs. These foods are also considered acidic foods, and the pH of the meat is between 5.5 and 6 [47]. BPA levels are expected to be higher in people who eat a protein-rich diet because more BPA can be leached into acidic foods [48,49]. Patients on a free diet are more likely to store protein-rich foods in plastic food containers than those on a Phe-restricted diet and are therefore more likely to be exposed to BPA. No statistically significant difference was observed in BPA levels among individuals in the overall group when analyzed according to their usage of plastic food containers. Patients in the Phe-restricted diet group are less exposed to BPA as they consume less protein-rich foods in the acidic food group. Therefore, BPA exposure via “plastic food containers” will be lower in the general group through the mechanism mentioned above. Despite the assertion in the methodology section that datasets with fewer than 10 cases would not be subjected to analysis, no statistically significant difference was identified when plasma BPA levels in the Phe-restricted diet group were compared between those who “use plastic storage containers” (n = 27) and those who did not (n = 7) (*p* = 0.478). In addition, the median BPA level of those using “plastic food containers” in the free-diet group was higher than the median BPA level of those using “plastic food containers” in the overall group (29.88 vs. 21.61 ng/mL). These data also support our idea above.

With the increasing concerns about BPA in the scientific world and society, and the involvement of regulators, the plastics industry has started to use a series of bisphenol derivatives, including BPF, called “bisphenol analogs” as alternatives to BPA. BPF is most commonly used in plastics for thickening and strengthening but has also been found in personal care products, coatings, and many foods [30]. Although BPA is still the most commonly detected bisphenol derivative in previous studies, the frequency of detection of bisphenol analogs is increasing [50,51]. When we examined the effect of participant characteristics on BPF levels, it was found to be lower in those who used plastic shower curtains, both in the general group and in those who did not diet (*p* = 0.013 and *p* = 0.01, respectively). This was the opposite of what was expected. Otherwise, we found no relationship between participant characteristics and BPF.

### 4.2. Phthalates

In the present study, the median levels of DBP, DEHP, and MEHP were 0.52, 0.48, and 0.23 ng/mL, respectively. In a study conducted in Türkiye by Kardas et al., mean plasma DEHP and MEHP values in healthy children with an average age of 7.5 were measured as 1.62 µg/mL and 0.29 µg/mL, respectively [52]. Similar values have been reported in other studies from Türkiye [39,53]. The main source of phthalate exposure in children is diet, which accounts for approximately 90% of exposure in children aged 3–11 years [11]. When we examined the levels of phthalates, especially DEHP and MEHP, according to the dietary treatment status of the patients, higher exposures were found in those who did not receive dietary treatment, but this did not reach statistical significance, probably due to the small number of cases (DEHP: 0.41 ng/mL vs. 0.56 ng/mL, *p* = 0.086, MEHP: 0.42 ng/mL vs. 0.54 ng/mL, *p* = 0.092 for dieters and non-dieters, respectively). For the reasons mentioned above, younger children are more exposed to EDCs. Consistent with this fact, plasma MEHP levels were higher in children younger than 4 years (*p* = 0.027) in our study, and there was a borderline association with DBP exposure (0.32 ng/mL for <4 years vs. 0.19 ng/mL for ≥4 years, *p* = 0.065). In the overall population, MEHP levels were higher in those using plastic food containers and plastic shower curtains, and this was an expected result. The presence of plastic tablecloths caused an increase in DBP and DEHP, while the use of bottled water was associated with an increase in DBP. Since the flexibility properties of DEHP and DBP are different, the amounts used in plastics used for bottled water, tablecloths, and shower curtains vary depending on the desired degree of flexibility [11,54]. This explains why different phthalate exposures are associated with different types of plastics. In 2015, Türkiye restricted the use of phthalates, including DBP and DEHP, in toys, in line with regulations of the EU. As of 1 January 2024, these restrictions have been expanded to electrical and electronic equipment [55]. As a result, we did not find a relationship between plastic toys and bisphenol and phthalates in all groups. One of the most interesting findings of our study was that no association was observed between the participant characteristics and phthalates in the Phe-restricted diet group. In the free-diet group, consistent with the literature, DBP levels were higher in those using bottled water and MEHP levels were higher in those using canned foods and plastic shower curtains. It is possible that health literacy is higher among parents of patients on Phe-restricted diets because they have a “disease that requires around-the-clock dietary control” to manage. This may have led parents to contribute to other healthy lifestyle behaviors and, if they used plastic, to prefer relatively safer plastics (BPA-free, phthalate-free, etc.) and/or to use them less frequently. Many studies in the literature have reported that there is a strong linear relationship between education level and health literacy [56,57]. However, inconsistent with this situation, we observed that plasma MEHP levels increased as the father’s education level increased. 

### 4.3. Strengths and Limitation

This study is the first in the literature to examine plasticizer levels in patients with PKU based on their dietary treatment. The sources of environmental exposures were comprehensively questioned, and many factors were analyzed together. The simultaneous assessment of more than one plasticizer has shown that exposures to plasticizers that are usually classified in the same group may differ. The main limitation of our study was the small number of participants. The fact that PKU is a “rare disease” was the main factor limiting the number of participants. Another limitation was that only one sample could be taken from the participants. If more samples in a period of time could have been taken at certain intervals, we can postulate that the relationship we found would have been stronger. 

## 5. Conclusions

In conclusion, we observed that PKU patients on a free diet had higher exposure to certain plasticizers. The differences in exposure routes to environmental EDCs between groups receiving dietary treatment or not highlight the importance of diet. Further research is needed to prevent PKU patients from having their growth and development risks exacerbated by environmental EDCS due to their underlying pathological condition.

## Figures and Tables

**Table 1:** General characteristics of the study subjects and paternal data.

*Sex, boy* *	49 (46.7)	*Maternal chronic disease* *	17 (16.2)
*Age, months* **	45.3 ± 13.4	*Paternal age, year* **	35.4 ± 4.9
*Age, <4 years* *	59 (56.2)	*Paternal education level* *	
*BAZ* *		≤8 years	64 (61.0)
≤−1	11 (10.5)	>8 years	41 (39.0)
−1< <1	64 (61.0)	*Paternal chronic disease* *	12 (11.4)
≥1 SDS	30 (28.6)	*Parent’s smoking status* *	
*Birth week* *		None of	40 (38.1)
Preterm	11 (10.5)	Only mother	5 (4.8)
Term/postterm	94 (89.5)	Only father	40 (38.1)
*Birth weight, g* **	3218 ± 570.2	Mother and father	20 (19.0)
*Type of birth* *		*Settlement* *	
Vaginal delivery	35 (33.3)	Rural	9 (8.6)
C/S	70 (66.7)	Urban/semi-urban	96 (91.4)
*Diagnosis* *		*Family structure* *	
HPA	38 (36.2)	Nuclear	89 (84.8)
PKU	30 (28.6)	Extended	16 (15.2)
BH4 responsive	37 (35.2)	*Perception of economic level* *	
*Time of diagnosis, day* ***	18 (11.0–25.5)	Income is less than expenses	33 (31.4)
*Treatment* *		Income is equal to expenses	53 (50.5)
PA-restricted diet	34 (32.4)	Income is more than expenses	19 (18.1)
BH4	34 (32.4)	*Sources of plastic exposure* *	
No need for treatment	37 (35.2)	Plastic baby bottle	21 (20.0)
*Phe-restricted diet* *		Baby shampoo	94 (89.5)
Yes	34 (32.4)	Plastic food container	74 (70.5)
No	71 (67.6)	Plastic water heater	19 (18.1)
*Diagnosis Phe* ***	6.2 (3.25–20.94)	Frozen food	44 (41.9)
*Mean Phe* ***	3.06 (2.36–4.10)	Canned food	57 (54.3)
*Mean Phe* *		Packaged food	101 (96.2)
<6 mg/dL	96 (91.4)	Canned beverage	40 (38.1)
≥6 mg/dL	9 (8.6)	PVC	95 (90.5)
*Birth order* *		Plastic shower curtain	31 (29.5)
First child	50 (47.6)	Plastic tablecloth	72 (68.6)
≥2. child	55 (52.4)	Cartridge printer	9 (8.6)
*Number of total children* *		Room freshener/spray/deodorant	49 (46.7)
1	43 (41.0)	Buying a new carpet in the last year	18 (17.1)
2	38 (36.2)	Buying a new furniture in the last year	24 (22.9)
3 and more	24 (22.9)	Using plastic containers when preparing and/or eating food	56 (53.3)
*Total breastfeeding time, month* ***	16.0 (6.0–24.0)	* Water used for cooking and/or drinking*	
*Total breastfeeding time* *		Tap water	64 (61.0)
0–5 ay	24 (22.9)	Spring water	14 (13.3)
6–11	22 (21.0)	Bottled water	39 (37.1)
12–23	29 (27.6)	Purified water	30 (28.6)
24 ay and more	39 (28.6)	* Toys*	
*Maternal age, year* **	32.1 ± 5.1	Plastic	103 (98.1)
*Maternal education level* *		Painted wood	44 (41.9)
≤8 years	63 (60.0)	Unpainted wood	59 (56.2)
>8 years	42 (40.0)	Plush	88 (83.8)

* n, %; ** Mean ± SD; *** Median (IQR). BAZ: BMI for age z-score, C/S: Cesarean section, HPA: hyperphenylalaninemia (blood phenylalanine levels within 2–6 mg/dL without any treatment), PKU: phenylketonuria, BH4: tetrahydrobiopterin, Phe: phenylalanine, PVC: polyvinyl chloride.

**Table 2:** Distribution of plasma BPA, BPF, MEHP, DEHP, and DBP levels.

	Percentiles					
	GMT	10	25	50	75	90
BPA. ng/mL	20.68	8.35	12.97	20.13	38.79	50.00
BPF, ng/mL	4.80	1.00	3.04	6.06	8.87	17.89
MEHP, ng/mL	0.46	0.12	0.33	0.52	0.82	1.30
DEHP, ng/mL	0.46	0.24	0.31	0.48	0.73	0.97
DBP, ng/mL	0.21	0.04	0.09	0.23	0.49	0.81

BPA: bisphenol A, BPF: bisphenol F, DBP: di-butyl phthalate, DEHP: di(2-ethylhexyl) phthalate, MEHP: mono-(2-ethylhexyl) phthalate.

**Table 3:** Relationship between participant characteristics and plasma BPA, BPF, DBP, DEHP, and MEHP levels.

	BPA (ng/mL)	BPF (ng/mL)	DBP (ng/mL)	DEHP (ng/mL)	MEHP (ng/mL)
*Sex*					
Girl (n = 56)	17.41 (12.86–30.59)	5.79 (1.76–8.26)	0.28 (0.11–0.52)	0.50 (0.31–0.75)	0.54 (0.32–0.77)
Boy (n = 49)	25.97 (12.97–41.84)	6.67 (3.50–10.42)	0.22 (0.08–0.43)	0.46 (0.31–0.72)	0.50 (0.33–0.95)
*p*	0.144	0.179	0.268	0.676	0.792
*Age*					
<4 years (n = 59)	17.46 (10.71–32.65)	5.78 (2.32–13.06)	0.32 (0.11–0.62)	0.50 (0.38–0.73)	0.56 (0.36–0.95)
≥4 years (n = 46)	24.96 (16.08–42.52)	6.17 (3.51–7.79)	0.19 (0.09–0.39)	0.46 (0.28–0.74)	0.43 (0.17–0.70)
*p*	0.084	0.696	0.065	0.349	0.027
*BAZ*					
≤−1 (n = 11)	25.97 (14.49–41.33)	4.33 (1.20–8.97)	0.34 (0.21–0.77)	0.51 (0.40–0.75)	0.57 (0.50–0.78)
−1< <1 (n = 64)	20.07 (12.85–32.35)	5.89 (3.03–9.28)	0.26 (0.09–0.46)	0.50 (0.31–0.72)	0.52 (0.26–0.74)
≥1 SDS (n = 30)	17.07 (11.36–46.69)	6.67 (3.35–8.10)	0.19 (0.09–0.50)	0.43 (0.28–0.74)	0.43 (0.34–1.13)
*p*	0.323	0.456	0.277	0.635	0.158
*Diagnosis*					
HPA (n = 38)	23.05 (12.96–40.36)	6.16 (2.16–12.87)	0.22 (0.08–0.49)	0.46 (0.36–0.72)	0.55 (0.34–0.99)
PKU (n = 30)	16.97 (11.33–30.07)	6.85 (4.11–7.82)	0.20 (0.11–0.50)	0.41 (0.30–0.63)	0.42 (0.20–0.74)
BH4 responsive (n = 37)	25.90 (15.12–40.97)	5.12 (3.04–8.92)	0.28 (0.11–0.50)	0.56 (0.30–0.76)	0.54 (0.38–0.77)
*p*	0.270	0.743	0.744	0.277	0.286
*Phe-restricted diet*					
Yes (n = 34)	17.71 (11.33–30.70)	6.85 (4.93–8.12)	0.20 (0.11–0.40)	0.41 (0.30–0.55)	0.42 (0.20–0.74)
No (n = 71)	25.51 (13.53–40.35)	5.44 (2.32–10.81)	0.28 (0.09–0.49)	0.56 (0.32–0.77)	0.54 (0.38–0.88)
*p*	0.267	0.413	0.489	0.086	0.092
*Birth order*					
First child (n = 50)	16.91 (12.46–31.09)	5.79 (2.28–8.10)	0.23 (0.09–0.49)	0.48 (0.30–0.81)	0.55 (0.33–0.91)
≥2. child (n = 55)	22.88 (13.20–40.61)	6.67 (3.31–9.24)	0.22 (0.09–0.49)	0.48 (0.32–0.68)	0.50 (0.32–0.74)
*p*	0.203	0.468	0.944	0.775	0.294
*Number of total children*					
1 (n = 43)	16.57 (11.39–30.69)	5.24 (1.97–7.45)	0.23 (0.09–0.49)	0.45 (0.30–0.81)	0.56 (0.34–0.93)
2 (n = 38)	23.02 (14.17–40.36)	6.16 (3.71–9.28)	0.21 (0.10–0.47)	0.47 (0.27–0.67)	0.54 (0.34–0.78)
3 and more (n = 24)	20.86 (12.67–41.37)	6.93 (3.54–9.07)	0.28 (0.12–0.53)	0.53 (0.40–0.70)	0.40 (0.15–0.59)
*p*	0.425	0.384	0.716	0.607	0.144
*Family structure*					
Nuclear (n = 89)	18.88 (11.37–37.48)	6.07 (3.19–8.50)	0.22 (0.09–0.46)	0.47 (0.31–0.75)	0.51 (0.28–0.84)
Extended (n = 16)	29.92 (17.21–40.83)	4.46 (1.64–11.99)	0.39 (0.15–0.77)	0.50 (0.33–0.56)	0.57 (0.41–0.73)
*p*	0.126	0.748	0.132	0.786	0.535
*Perception of economic level*					
Income is less than expenses (n = 33)	21.37 (13.49–36.49)	6.83 (2.38–10.04)	0.21 (0.10–0.40)	0.53 (0.32–0.71)	0.54 (0.34–0.80)
Income is equal to expenses (n = 53)	18.88 (12.41–39.75)	5.79 (3.53–8.39)	0.27 (0.09–0.67)	0.48 (0.37–0.74)	0.43 (0.20–0.83)
Income is more than expenses (n = 19)	20.00 (13.53–34.79)	5.80 (1.38–7.79)	0.22 (0.09–0.46)	0.32 (0.28–0.71)	0.60 (0.46–0.86)
*p*	0.878	0.616	0.624	0.627	0.230
*Total breastfeeding time, month*					
0–5 (n = 24)	25.12 (16.40–38.17)	6.81 (1.95–7.96)	0.24 (0.15–0.49)	0.48 (0.33–0.70)	0.52 (0.35–0.75)
6–11 (n = 22)	16.99 (11.05–30.50)	6.27 (4.19–7.72)	0.28 (0.11–0.64)	0.53 (0.35–0.82)	0.50 (0.31–0.65)
12–23 (n = 29)	25.51 (14.24–44.81)	4.65 (1.39–9.01)	0.27 (0.11–0.49)	0.48 (0.28–0.74)	0.50 (0.35–0.85)
24 and more (n = 39)	20.95 (13.00–31.44)	6.28 (2.96–13.49)	0.19 (0.06–0.44)	0.47 (0.26–0.74)	0.57 (0.15–0.97)
*p*	0.406	0.654	0.531	0.768	0.921
*Maternal education level*					
≤8 years (n = 64)	21.37 (12.98–39.16)	6.07 (3.56–8.55)	0.23 (0.11–0.46)	0.50 (0.32–0.73)	0.50 (0.34–0.69)
>8 years (n = 41)	17.07 (12.72–36.18)	5.91 (1.83–9.04)	0.22 (0.09–0.53)	0.46 (0.29–0.78)	0.53 (0.31–1.13)
*p*	0.649	0.587	0.761	0.771	0.147
*Maternal chronic disease*					
Yes (n = 17)	16.81 (12.04–30.28)	6.07 (1.20–7.91)	0.18 (0.09–0.34)	0.43 (0.29–0.70)	0.51 (0.33–0.92)
No (n = 88)	20.44 (12.99–40.05)	6.05 (3.33–9.17)	0.27 (0.09–0.53)	0.50 (0.31–0.73)	0.53 (0.32–0.78)
*p*	0.441	0.523	0.237	0.495	0.852
*Paternal education level*					
≤8 years (n = 64)	22.12 (12.85–40.05)	6.61 (3.86–8.92)	0.23 (0.10–0.48)	0.50 (0.32–0.75)	0.53 (0.34–0.73)
>8 years (n = 41)	17.46 (13.08–33.72)	5.24 (1.37–8.95)	0.23 (0.09–0.51)	0.46 (0.29–0.72)	0.52 (0.32–1.06)
*p*	0.966	0.136	0.987	0.625	0.273
*Paternal chronic disease*					
Yes (n = 12)	18.90 (12.40–38.12)	6.17 (1.80–11.56)	0.18 (0.08–0.29)	0.49 (0.30–0.63)	0.51 (0.25–0.71)
No (n = 93)	20.13 (12.97–38.79)	6.05 (3.19–8.65)	0.25 (0.09–0.51)	0.48 (0.31–0.76)	0.53 (0.33–0.85)
*p*	0.793	0.948	0.295	0.478	0.669
*Sources of plastic exposure*					
*Plastic baby bottle*					
Yes (n = 21)	17.12 (9.75–44.00)	6.06 (2.24–8.25)	0.29 (0.13–0.48)	0.46 (0.34–0.69)	0.54 (0.30–1.13)
No (n = 84)	20.44 (13.28–37.95)	6.06 (3.13–8.92)	0.22 (0.09–0.49)	0.49 (0.30–0.75)	0.51 (0.32–0.77)
*p*	0.718	0.823	0.671	0.997	0.662
*Baby shampoo*					
Yes (n = 94)	19.81 (12.61–38.60)	6.05 (2.96–8.58)	0.26 (0.09–0.53)	0.50 (0.33–0.75)	0.52 (0.34–0.89)
No (n = 11)	20.13 (15.54–40.35)	6.95 (3.07–9.07)	0.16 (0.08–0.27)	0.29 (0.21–0.48)	0.53 (0.13–0.62)
*p*	0.762	0.818	0.225	0.014	0.303
*Plastic food container*					
Yes (n = 74)	21.61 (13.00–40.79)	5.79 (2.80–8.81)	0.24 (0.10–0.51)	0.49 (0.30–0.74)	0.56 (0.38–0.91)
No (n = 31)	18.88 (10.71–30.38)	6.67 (3.07–10.81)	0.22 (0.09–0.46)	0.45 (0.32–0.71)	0.39 (0.25–0.57)
*p*	0.209	0.276	0.511	0.952	0.019
*Plastic water heater*					
Yes (n = 19)	17.12 (12.81–41.33)	5.24 (3.37–7.25)	0.22 (0.10–0.49)	0.45 (0.30–0.75)	0.60 (0.26–0.91)
No (n = 86)	20.44 (12.97–38.60)	6.18 (2.69–9.28)	0.24 (0.09–0.49)	0.49 (0.31–0.73)	0.51 (0.34–0.76)
*p*	0.752	0.375	0.662	0.768	0.641
*Frozen food*					
Yes (n = 44)	25.73 (12.97–38.62)	6.17 (3.37–8.42)	0.26 (0.10–0.60)	0.52 (0.32–0.70)	0.52 (0.26–0.88)
No (n = 61)	18.88 (12.50–38.79)	5.80 (2.91–9.02)	0.21 (0.08–0.45)	0.47 (0.30–0.79)	0.53 (0.34–0.76)
*p*	0.568	0.738	0.268	0.961	0.997
*Canned food*					
Yes (n = 57)	20.35 (13.00–39.76)	6.05 (3.46–9.21)	0.23 (0.09–0.48)	0.50 (0.30–0.72)	0.56 (0.40–0.84)
No (n = 48)	19.63 (12.25–37.95)	6.29 (1.59–8.42)	0.22 (0.09–0.52)	0.46 (0.31–0.75)	0.40 (0.21–0.76)
*p*	0.967	0.545	0.918	0.817	0.099
*Canned beverage*					
Yes (n = 40)	20.44 (12.85–47.32)	6.05 (2.39–8.68)	0.22 (0.10–0.43)	0.49 (0.30–0.66)	0.48 (0.25–0.61)
No (n = 65)	20.00 (13.00–2.33)	6.07 (3.19–9.02)	0.27 (0.09–0.53)	0.47 (0.31–0.81)	0.56 (0.34–0.91)
*p*	0.245	0.864	0.682	0.598	0.147
*PVC*					
Yes (n = 95)	21.37 (12.98–40.35)	5.99 (3.01–9.07)	0.22 (0.09–0.49)	0.50 (0.30–0.75)	0.54 (0.34–0.86)
No (n = 10)	16.83 (11.83–22.64)	6.69 (4.35–8.47)	0.28 (0.10–0.69)	0.43 (0.31–0.58)	0.37 (0.20–0.75)
*p*	0.221	0.836	0.552	0.499	0.300
*Plastic shower curtain*					
Yes (n = 31)	25.90 (13.20–41.33)	4.07 (1.25–6.83)	0.11 (0.05–0.62)	0.53 (0.30–0.75)	0.57 (0.40–1.11)
No (n = 74)	20.07 (11.38–37.01)	6.67 (4.14–9.11)	0.26 (0.12–0.46)	0.46 (0.31–0.71)	0.47 (0.25–0.75)
*p*	0.399	0.013	0.261	0.684	0.014
*Plastic tablecloth*					
Yes (n = 72)	19.63 (12.22–40.05)	6.02 (2.86–8.50)	0.29 (0.12–0.54)	0.51 (0.39–0.75)	0.55 (0.32–0.95)
No (n = 33)	21.37 (15.13–35.39)	6.27 (3.19–9.17)	0.11 (0.08–0.31)	0.32 (0.26–0.72)	0.46 (0.31–0.63)
*p*	0.622	0.691	0.040	0.049	0.166
*Room freshener/spray/deodorant*					
Yes (n = 49)	18.29 (12.89–41.00)	6.27 (3.62–8.87)	0.22 (0.11–0.51)	0.45 (0.32–0.67)	0.56 (0.28–0.90)
No (n = 56)	20.24 (13.02–34.25)	6.03 (2.20–9.07)	0.24 (0.07–0.48)	0.53 (0.28–0.79)	0.51 (0.34–0.75)
*p*	0.780	0.632	0.428	0.502	0.908
*Buying new carpet in the last year*					
Yes (n = 18)	22.22 (15.32–33.62)	5.14 (1.11–7.97)	0.24 (0.09–0.45)	0.43 (0.37–0.76)	0.63 (0.32–0.97)
No (n = 87)	20.13 (12.81–40.35)	6.28 (3.07–9.07)	0.23 (0.09–0.49)	0.50 (0.30–0.73)	0.51 (0.32–0.78)
*p*	0.915	0.296	0.747	0.848	0.454
*Buying new furniture in the last year*					
Yes (n = 24)	19.70 (12.23–39.41)	6.05 (4.65–9.02)	0.23 (0.12–0.35)	0.43 (0.32–0.77)	0.68 (0.38–1.04)
No (n = 81)	20.35 (12.97–38.79)	6.07 (2.24–8.87)	0.23 (0.09–0.54)	0.50 (0.30–0.73)	0.45 (0.32–0.75)
*p*	0.991	0.332	0.480	0.879	0.068
*Using plastic when preparing and/or eating food*					
Yes (n = 56)	17.41 (13.03–36.10)	6.39 (3.96–8.71)	0.27 (0.11–0.46)	0.47 (0.30–0.66)	0.44 (0.21–0.75)
No (n = 49)	25.90 (12.18–41.71)	5.24 (1.93–9.94)	0.21 (0.08–0.49)	0.52 (0.31–0.77)	0.56 (0.39–0.94)
*p*	0.362	0.450	0.639	0.409	0.077
*Kind of water used for cooking and/or drinking*					
Tap water					
Yes (n = 64)	20.33 (13.28–38.50)	6.41 (1.81–12.16)	0.25 (0.09–0.54)	0.47 (0.30–0.76)	0.53 (0.38–0.90)
No (n = 41)	19.26 (11.70–39.87)	5.99 (3.76–7.23)	0.22 (0.10–0.45)	0.50 (0.31–0.68)	0.46 (0.15–0.75)
*p*	0.785	0.468	0.484	0.698	0.167
Spring water					
Yes (n = 14)	36.97 (16.34–44.70)	8.09 (2.79–15.63)	0.22 (0.08–0.56)	0.53 (0.38–0.74)	0.45 (0.14–0.69)
No (n = 91)	18.88 (12.02–32.37)	6.05 (3.01–8.35)	0.23 (0.09–0.46)	0.47 (0.30–0.73)	0.53 (0.34–0.88)
*p*	0.053	0.272	0.959	0.749	0.327
Bottled water					
Yes (n = 39)	18.88 (12.02–30.84)	6.05 (2.32–7.81)	0.33 (0.18–0.75)	0.52 (0.40–0.77)	0.52 (0.34–1.03)
No (n = 66)	20.44 (13.15–41.34)	6.17 (3.05–9.25)	0.16 (0.08–0.41)	0.47 (0.29–0.73)	0.51 (0.27–0.74)
*p*	0.442	0.850	0.010	0.143	0.171
Purified water					
Yes (n = 30)	20.86 (10.63–39.15)	6.03 (3.05–7.57)	0.18 (0.06–0.36)	0.46 (0.28–0.80)	0.48 (0.15–0.71)
No (n = 75)	20.00 (13.20–39.16)	6.06 (2.32–9.24)	0.27 (0.11–0.54)	0.50 (0.33–0.71)	0.53 (0.34–0.86)
*p*	0.831	0.681	0.082	0.804	0.297
*Toys*					
Painted wood					
Yes (n = 44)	17.07 (11.29–32.56)	5.78 (2.44–7.96)	0.19 (0.07–0.52)	0.44 (0.32–0.75)	0.48 (0.28–0.93)
No (n = 61)	25.51 (13.49–40.49)	6.51 (3.34–9.25)	0.27 (0.11–0.48)	0.50 (0.30–0.71)	0.54 (0.35–0.75)
*p*	0.268	0.473	0.320	0.979	0.738
Unpainted wood					
Yes (n = 59)	21.37 (14.73–41.33)	6.05 (3.07–8.55)	0.21 (0.09–0.49)	0.50 (0.30–0.73)	0.56 (0.28–0.86)
No (n = 46)	19.07 (9.98–32.39)	6.06 (2.61–9.63)	0.26 (0.11–0.44)	0.45 (0.31–0.71)	0.50 (0.34–0.70)
*p*	0.174	0.923	0.647	0.654	0.642
Plush					
Yes (n = 88)	18.77 (12.96–36.10)	6.06 (3.14–9.20)	0.27 (0.10–0.53)	0.50 (0.31–0.75)	0.53 (0.32–0.81)
No (n = 17)	29.90 (13.81–45.90)	6.05 (1.85–8.13)	0.15 (0.08–0.31)	0.43 (0.26–0.70)	0.39 (0.33–1.01)
*p*	0.283	0.617	0.093	0.478	0.572

BAZ: BMI for age z-score, HPA: hyperphenylalaninemia (blood phenylalanine levels within 2–6 mg/dL without any treatment), PKU: phenylketonuria, BH4: tetrahydrobiopterin, Phe: phenylalanine, PVC: polyvinyl chloride. A *p*-value of less than 0.05 was considered to be statistically significant.

**Table 4:** The association between diet type and upper tertiles of bisphenols and phthalate metabolites after adjusting child characteristics (Model 1) and adjusting both child characteristics and exposure sources (Model 2).

			Model 1 ^&^		Model 2 ^#^	
	% *	*p* ^$^	AOR	95% CI	AOR	95% CI
Dependent variable: BPA > 31 ng/mL		0.098				
Phe-restricted diet	20.6		1.00		1.00	
Free diet	36.6		3.34	1.09–10.25	18.64	2.09—166.42
Dependent variable: BPF > 7.5		0.345				
Phe-restricted diet	32.4		1.00		1.00	
Free diet	33.8		0.93	0.34–2.52	0.57	0.12–2.79
Dependent variable: DBP > 0.70 ng/mL		0.180				
Phe-restricted diet	23.5		1.00		1.00	
Free diet	36.6		2.52	0.88–7.20	5.06	0.88–29.04
Dependent variable: DEHP > 0.65 ng/mL		0.055				
Phe-restricted diet	20.6		1.00		1.00	
Free diet	39.4		3.01	1.06–8.60	2.82	0.79–10.16
Dependent variable: MEHP > 0.40 ng/mL		0.449				
Phe-restricted diet	26.5		1.00		1.00	
Free diet	33.8		1.00	0.35–2.83	1.48	0.22–10.06

* row percentage; ^$^ Chi-square test. ^&^ adjusted for age, gender, BMI for age z-score (BAZ), birth order and total breastfeeding duration; ^#^ adjusted for Model 1 and exposure sources. AOR: adjusted odds ratio, BPA: bisphenol A, BPF: bisphenol F, CI: confidence interval, DBP: di-buthyl phthalate, DEHP: di-ethylhexyl phthalate, MEHP: mono-ethylhexyl phthalate, Phe: phenylalanine.

## Data Availability

Due to privacy, the data presented in this study are available on request from the corresponding author.

## Supplementary Materials

The following supporting information can be downloaded at: https://www.mdpi.com/article/10.3390/nu16183213/s1, Table S1: Relationship between participant characteristics and plasma BPA, BPF, MEHP, DEHP and DBP levels in the PA-restricted diet group; Table S2: Relationship between participant characteristics and plasma BPA, BPF, MEHP, DEHP and DBP levels in free diet group.
