# Supplementary material for: Evaluation of Exposure to Bisphenol A, Bisphenol F, and Phthalates in Patients with Phenylketonuria and Its Differences According to Dietary Status
Source: Nutrients. 2024 Sep 23;16(18):3213. doi: 10.3390/nu16183213 (PMC11435359; doi:10.3390/nu16183213)
Supplement: Supplementary file 1 [file nutrients-16-03213-s001.zip › nutrients-3189491-supplementary.pdf]

Table S1. Relationship between participant characteristics and plasma BPA, BPF, MEHP, DEHP and DBP levels in the PA-restricted diet group.

	BPA (ng/mL)	BPF (ng/mL)	DBP (ng/mL)	DEHP (ng/mL)	MEHP (ng/mL)
Sex					
Girl (n=18)	16.69 (9.94-30.09)	7.09 (3.37-8.20)	0.20 (0.12-0.36)	0.40 (0.28-0.49)	0.55 (0.13-0.84)
Boy (n=16)	18.58 (12.25-39.47)	6.80 (5.55-8.15)	0.20 (0.09-0.49)	0.49 (0.31-0.67)	0.37 (0.20-0.54)
p	0.242	0.286	0.197	0.203	0.183
Age					
<4 years (n=17)	12.96 (10.00-25.13)	6.67 (2.07-7.60)	0.21 (0.13-0.56)	0.43 (0.34-0.65)	0.54 (0.29-0.88)
≥4 years (n=17)	20.13 (16.07-36.59)	6.92 (5.58-8.60)	0.18 (0.07-0.27)	0.36 (0.27-0.52)	0.34 (0.12-0.58)
p	0.170	0.339	0.150	0.182	0.131
Birth order					
First child (n=14)	16.06 (11.05-25.03)	7.00 (4.83- 8.32)	0.17 (0.11-0.32)	0.36 (0.29-0.61)	0.39 (0.18-0.83)
≥2. child (n=20)	20.24 (11.52-30.79)	6.75 (4.50-8.27)	0.24 (0.12-0.46)	0.42 (0.32-0.60)	0.54 (0.19-0.71)
p	0.323	0.569	0.377	0.616	0.877
Number of total children					
1 (n=11)	12.96 (10.40-18.88)	7.14 (3.84-8.01)	0.15 (0.08-0.53)	0.31 (0.29-0.52)	0.40 (0.12-1.06)
2 (n=13)	20.13 (13.78-37.28)	6.67 (2.51-8.13)	0.19 (0.11-0.29)	0.38 (0.26-0.67)	0.35 (0.20-0.67)
3 and more (n=10)	19.32 (10.92-34.55)	7.09 (5.79-8.65)	0.28 (0.17-0.52)	0.44 (0.40-0.63)	0.55 (0.17-0.82)
p	0.325	0.523	0.379	0.422	0.875
Maternal education level					
≤8 years (n=23)	19.26 (11.35-30.84)	6.92 (5.79-8.01)	0.21 (0.12-0.37)	0.42 (0.30-0.62)	0.40 (0.18-0.74)
>8 years (n=11)	15.54 (11.26-20.13)	5.40 (2.16-8.76)	0.18 (0.08-0.53)	0.40 (0.29-0.53)	0.45 (0.20-0.75)
p	0.188	0.363	0.800	0.942	0.885
Paternal education level					
≤8 years (n=23)	18.88 (10.40-30.66)	7.25 (5.99-8.45)	0.21 (0.11-0.49)	0.43 (0.30-0.62)	0.56 (0.20-0.77)
>8 years (n=11)	16.57 (11.35-42.34)	5.40 (1.97-7.14)	0.18 (0.08-0.32)	0.38 (0.29-0.53)	0.31 (0.13-0.46)
p	0.856	0.098	0.586	0.513	0.153
Plastic baby bottle					
Yes (n=13)	17.12 (10.48-44.00)	6.83 (3.97-12.04)	0.29 (0.16-0.57)	0.46 (0.40-0.67)	0.45 (0.19-0.88)
No (n=21)	18.88 (11.33-30.23)	6.86 (4.69-7.88)	0.15 (0.09-0.27)	0.39 (0.19-0.67)	0.39 (0.19-0.67)
p	0.834	0.807	0.104	0.055	0.675
Frozen food					
Yes (n=17)	17.12 (10.43-30.70)	6.76 (3.00-7.76)	0.25 (0.17-0.58)	0.43 (0.34-0.58)	0.45 (0.19-0.67)
No (n=17)	18.29 (11.37-37.28)	7.25 (5.28-8.92)	0.12 (0.09-0.29)	0.38 (0.27-0.59)	0.40 (0.17-0.76)
p	0.865	0.474	0.041	0.274	0.973
Canned food					
Yes (n=14)	19.50 (11.05-42.74)	7.29 (4.93-8.84)	0.17 (0.10-0.40)	0.40 (0.30-0.49)	0.43 (0.12-0.60)
No (n=20)	16.71 (11.36-30.40)	6.72 (4.23-7.50)	0.21 (0.12-0.49)	0.42 (0.29-0.65)	0.40 (0.20-0.76)

p	0.478	0.323	0.478	0.522	0.616
Canned beverage					
Yes (n=19)	17.12 (11.35-30.56)	6.86 (5.40-8.01)	0.19 (0.11-0.37)	0.46 (0.31-0.62)	0.40 (0.18-0.60)
No (n=15)	18.88 (10.40-30.84)	6.67 (4.21-9.07)	0.21 (0.11-0.53)	0.40 (0.26-0.52)	0.53 (0.31-0.77)
P	0.918	0.891	0.758	0.271	0.271
Room freshener/spray/ deodorant					
Yes (n=19)	27.04 (11.26-43.91)	7.25 (5.79-8.76)	0.21 (0.12-0.53)	0.42 (0.31-0.52)	0.40 (0.12-0.77)
No (n=15)	16.61 (11.39-20.13)	5.99 (1.97-7.45)	0.18 (0.07-0.32)	0.40 (0.25-0.80)	0.45 (0.26-0.74)
p	0.286	0.056	0.336	0.656	0.656
Using plastic when preparing and/or eating food					
Yes (n=22)	17.71 (11.11-44.35)	7.06 (5.69-8.88)	0.20 (0.11-0.41)	0.43 (0.31-0.63)	0.29 (0.12-0.64)
No (n=12)	17.74 (11.29-27.51)	6.23 (3.93-7.38)	0.20 (0.07-0.44)	0.36 (0.27-0.51)	0.54 (0.40-1.28)
p	0.444	0.204	0.709	0.292	0.063
Water used for cooking and/or drinking					
Tap water					
Yes (n=17)	20.13 (11.35-43.91)	7.25 (4.21-9.07)	0.21 (0.11- 0.37)	0.43 (0.29- 0.53)	0.56 (0.18- 0.77)
No (n=17)	16.57 (11.26- 27.04)	6.67 (5.17 -7.45)	0.19 (0.11- 0.49)	0.40 (0.30- 0.66)	0.35 (0.20- 0.56)
P	0.357	0.838	0.734	0.274	0.610
Bottled water					
Yes (n=12)	16.57 (11.70- 38.25)	5.40 (3.00- 7.11)	0.19 (0.13- 0.47)	0.40 (0.34-0.52)	0.39 (0.28-1.21)
No (n=22)	18.29 (10.83-30.61)	7.14 (5.79-8.60)	0.21 (0.11- 0.43)	0.43 (0.29-0.64)	0.46 (0.12- 0.67)
p	0.511	0.383	0.958	0.958	0.845
Toys					
Painted wood					
Yes (n=11)	16.57 (11.26- 20.13)	5.79 (4.21 -7.14)	0.15 (0.08-0.21)	0.40 (0.3-0.43)	0.34 (0.20- 0.53)
No (n=23)	19.26 (11.39-42.34)	7.25 (5.78-8.76)	0.25 (0.11-0.53)	0.46 (0.29-0.66)	0.54 (0.18-0.75)
p	0.274	0.176	0.091	0.612	0.383
Unpainted wood					
Yes (n=18)	18.74 (12.72-44.35)	6.80 (2.12-8.53)	0.20 (0.11-0.50)	0.41 (0.29-0.52)	0.40 (0.17-0.75)
No (n=16)	17.45 (10.02-25.31)	7.03 (5.78-7.89)	0.20 (0.09-0.36)	0.41 (0.30-0.67)	0.43 (0.22-0.59)
p	0.224	0.670	0.746	0.484	0.986

BPA: bisphenol A, BPF: bisphenol F, DBP: di-buthyl phthalate, DEHP: di-ethylhexyl phthalate, MEHP: mono-ethylhexyl phthalate, PA: phenylalanine, PVC: polyvinyl chloride

PA-restricted diet group includes 34 subjects.

A p-value of less than 0.05 was considered to be statistically significant.

Table S2. Relationship between participant characteristics and plasma BPA, BPF, MEHP, DEHP and DBP levels in free diet group.

	BPA (ng/mL)	BPF (ng/mL)	DBP (ng/mL)	DEHP (ng/mL)	MEHP (ng/mL)
Sex					
Girl (n=38)	17.46 (13.86-33.18)	4.89 (1.40-8.50)	0.33 (0.10-0.65)	0.56 (0.42-0.78)	0.53 (0.34-0.77)
Boy (n=33)	30.69 (13.09-44.59)	6.05 (2.91-14.28)	0.23 (0.06-0.43)	0.46 (0.27-0.74)	0.57 (0.39-1.06)
p	0.242	0.286	0.197	0.203	0.183
Age					
<4 years (n=42)	23.02 (12.29-35.22)	5.18 (2.69-14.94)	0.34 (0.08-0.64)	0.56 (0.38-0.76)	0.57 (0.39-0.97)
≥4 years (n=29)	29.35 (15.89-45.45)	5.80 (1.54-6.87)	0.20 (0.09-0.43)	0.56 (0.28-0.79)	0.50 (0.31-0.79)
P	0.184	0.346	0.287	0.939	0.124
Birth order					
First child (n=36)	23.02 (13.14-31.90)	5.07 (1.49-7.96)	0.30 (0.09-0.49)	0.56 (0.34-0.81)	0.57 (0.36-0.93)
≥2. child (n=35)	30.38 (15.52-41.33)	6.06 (3.07-14.33)	0.22 (0.09-0.54)	0.56 (0.32-0.73)	0.45 (0.38-0.74)
p	0.277	0.320	0.739	0.713	0.198
Number of total children					
1 (n=32)	24.22 (13.14-31.90)	4.73 (1.39-6.73)	0.28 (0.09-0.48)	0.53 (0.38-0.86)	0.62 (0.36-0.93)
2 (n=25)	25.51 (13.84-40.36)	6.05 (3.50-12.72)	0.22 (0.08-0.68)	0.50 (0.30-0.68)	0.57 (0.43-1.03)
≥3 (n=14)	25.87 (14.36-43.23)	6.51 (2.79-17.65)	0.34 (0.09-0.60)	0.59 (0.30-0.74)	0.39 (0.14-0.53)
p	0.667	0.372	0.963	0.604	0.017
Family structure					
Nuclear (n=59)	22.88 (13.20-40.35)	5.80 (2.81-9.35)	0.23 (0.09-0.46)	0.56 (0.30-0.81)	0.54 (0.36-0.92)
Extended (n=12)	28.93 (14.84-40.83)	4.10 (1.64-11.99)	0.42 (0.17-0.92)	0.53 (0.46-0.67)	0.53 (0.39-0.65)
p	0.485	0.581	0.143	0.957	0.701
Perception of economic level					
Income is less than expenses (n=22)	26.38 (13.73-44.25)	6.06 (1.37-11.72)	0.16 (0.06 0.39)	0.57 (0.31-0.82)	0.44 (0.34-0.58)
Income is equal to expenses (n=36)	21.48 (13.03-38.97)	4.78 (3.33-14.54)	0.35 (0.09-0.77)	0.50 (0.39-0.75)	0.56 (0.30-0.93)
Income is more than expenses (n=13)	25.90 (14.13-41.87)	5.80 (1.37-8.95)	0.33 (0.10-0.54)	0.62 (0.27-0.92)	0.69 (0.51-0.91)
p	0.693	0.887	0.075	0.900	0.189
Total breastfeeding time, month					
0-5 (n=12)	29.91 (15.11-42.44)	4.68 (1.17-7.54)	0.28 (0.07-0.45)	0.59 (0.43-0.84)	0.42 (0.35-0.52)
6-11 (n=12)	21.68 (13.23-45.70)	5.01 (3.28-8.82)	0.47 (0.20-0.79)	0.56 (0.38-0.89)	0.56 (0.39-0.92)
12-23 (n=18)	29.08 (15.98-43.23)	4.39 (1.29-9.67)	0.30 (0.09-0.70)	0.59 (0.29-0.76)	0.57 (0.41-0.94)
24 and more (n=29)	21.37 (13.00-31.75)	6.28 (3.16-14.59)	0.20 (0.06-0.45)	0.47 (0.26-0.75)	0.57 (0.15-0.99)
p	0.648	0.403	0.172	0.565	0.320
Maternal education level					
≤8 years (n=40)	24.20 (13.25-40.05)	5.01 (3.13-11.32)	0.28 (0.07-0.46)	0.56 (0.38-0.75)	0.51 (0.38-0.69)
>8 years (n=31)	25.57 (13.53-40.37)	6.05 (1.38-9.35)	0.28 (0.09-0.53)	0.47 (0.28-0.81)	0.64 (0.34-1.26)

p	0.912	0.889	0.685	0.715	0.085
Paternal education level					
≤8 years (n=41)	29.35 (13.00-40.49)	5.80 (3.34-11.15)	0.28 (0.07-0.48)	0.56 (0.33-0.79)	0.50 (0.35-0.68)
>8 years (n=30)	18.73 (15.02-33.18)	4.90 (1.30-10.28)	0.26 (0.09-0.65)	0.50 (0.29-0.74)	0.75 (0.39-1.27)
p	0.930	0.364	0.744	0.684	0.021
Sources of plastic exposure					
Plastic food container					
Yes (n=47)	29.88 (14.49-41.39)	4.90 (1.69-9.35)	0.32 (0.09-0.62)	0.56 (0.33-0.81)	0.57 (0.40-0.92)
No (n=24)	17.41 (9.13-29.44)	6.53 (2.44-11.99)	0.22 (0.08-0.45)	0.48 (0.32-0.71)	0.44 (0.28-0.66)
p	0.041	0.391	0.412	0.519	0.058
Plastic water heater					
Yes (n=12)	23.03 (13.83-51.34)	4.26 (3.33-5.66)	0.22 (0.09-0.82)	0.43 (0.30-0.77)	0.73 (0.42-0.90)
No (n=59)	25.51 (13.20-39.16)	6.06 (1.69-12.39)	0.28 (0.08-0.49)	0.56 (0.33-0.77)	0.52 (0.36-0.82)
p	0.902	0.300	0.771	0.452	0.369
Frozen food					
Yes (n=27)	25.97 (13.53-41.33)	6.06 (3.31-14.24)	0.28 (0.09-0.75)	0.56 (0.28-0.73)	0.56 (0.38-0.91)
No (n=44)	20.27 (13.39-38.97)	5.28 (1.40-10.35)	0.28 (0.08-0.46)	0.50 (0.32-0.81)	0.54 (0.36-0.80)
p	0.394	0.488	0.804	0.740	0.785
Canned food					
Yes (n=43)	25.51 (13.01-39.16)	5.44 (3.31-10.81)	0.28 (0.09-0.49)	0.57 (0.32-0.77)	0.57 (0.43-0.88)
No (n=28)	24.43 (16.16-41.13)	5.44 (1.21-14.73)	0.25 (0.09-0.52)	0.47 (0.34-0.77)	0.40 (0.26 -0.88)
p	0.394	0.659	0.837	0.689	0.044
Canned beverage					
Yes (n=21)	36.54 (14.35-49.72)	4.90 (1.40-13.31)	0.22 (0.09-0.45)	0.56 (0.29-0.68)	0.52 (0.39-0.65)
No (n=50)	20.68 (13.15-32.44)	5.61 (2.69-9.07)	0.28 (0.08-0.55)	0.53 (0.36-0.81)	0.56 (0.35-0.93)
P	0.066	0.701	0.920	0.504	0.579
Plastic shower curtain					
Yes (n=25)	25.97 (13.36-40.24)	2.81 (1.20-6.53)	0.22 (0.04-0.57)	0.62 (0.35-0.81)	0.67 (0.44-1.22)
No (n=46)	22.12 (13.68-40.36)	6.18 (3.83-11.72)	0.29 (0.11-0.47)	0.49 (0.32-0.74)	0.51 (0.33-0.77)
P	0.718	0.011	0.496	0.496	0.017
Plastic tablecloth					
Yes (n=47)	25.57 (13.01-40.35)	5.44 (2.81-10.81)	0.36 (0.09-0.62)	0.56 (0.41-0.81)	0.57 (0.38-0.98)
No (n=24)	24.20 (15.08-45.50)	5.52 (1.28-10.96)	0.18 (0.09-0.38)	0.41 (0.26-0.73)	0.47 (0.30-0.63)
p	0.766	0.827	0.234	0.106	0.056
Room freshener/spray/ deodorant					
Yes (n=30)	17.32 (13.40-40.43)	4.78 (1.62-9.07)	0.27 (0.10-0.50)	0.53 (0.32-0.74)	0.60 (0.37-0.91)
No (n=41)	25.57 (14.73-39.75)	6.06 (2.57-13.65)	0.28 (0.07-0.55)	0.56 (0.35-0.79)	0.52 (0.37-0.80)
P	0.697	0.481	0.629	0.641	0.545
Buying a new carpet in the last year					
Yes (n=13)	25.57 (15.83-34.59)	4.90 (1.08-7.16)	0.28 (0.09-0.59)	0.46 (0.40-0.83)	0.75 (0.39-1.17)
No (n=58)	24.20	5.91	0.28	0.56	0.52

	(13.00-40.43)	(2.69-11.72)	(0.09-0.50)	(0.31-0.77)	(0.35-0.82)
P	0.911	0.384	0.994	0.829	0.117
Buying a new furniture in the last year					
Yes (n=15)	31.14 (16.26-40.61)	6.06 (4.56-18.38)	0.27 (0.11-0.40)	0.46 (0.32-0.70)	0.69 (0.51-0.98)
No (n=56)	20.95 (12.99-38.97)	4.89 (1.49-10.35)	0.30 (0.08-0.60)	0.56 (0.34-0.77)	0.47 (0.34-0.82)
P	0.202	0.155	0.554	0.637	0.053
Using plastic when preparing and/or eating food					
Yes (n=34)	17.41 (13.45-32.05)	5.93 (3.53-8.65)	0.33 (0.09-0.48)	0.50 (0.30-0.67)	0.52 (0.33-0.80)
No (n=37)	29.35 (13.49-48.85)	4.65 (1.39-13.36)	0.23 (0.08-0.52)	0.57 (0.35-0.81)	0.56 (0.38-0.94)
p	0.120	0.769	0.734	0.207	0.573
Water used for cooking and/or drinking					
Tap water					
Yes (n=47)	25.90 (13.53-39.16)	6.05 (1.42-14.24)	0.33 (0.09-0.62)	0.57 (0.38-0.82)	0.54 (0.40-0.92)
No (n=24)	24.20 (13.41-42.62)	4.45 (3.03-6.45)	0.23 (0.06-0.42)	0.50 (0.28-0.68)	0.55 (0.15-0.77)
p	0.589	0.408	0.231	0.293	0.395
Bottled water					
Yes (n=27)	25.57 (16.26-32.65)	6.05 (1.42-8.55)	0.40 (0.23-0.81)	0.62 (0.45-0.82)	0.68 (0.40-1.09)
No (n=44)	24.20 (13.28-41.37)	5.01 (2.86-11.32)	0.15 (0.06-0.43)	0.47 (0.28-0.75)	0.52 (0.34-0.73)
p	0.670	0.896	0.004	0.073	0.058
Purified water					
Yes (n=21)	25.51 (11.76-44.59)	5.12 (3.04-8.85)	0.16 (0.05-0.39)	0.47 (0.27-0.78)	0.54 (0.15-0.83)
No (n=50)	23.22 (13.86-39.46)	5.61 (1.41-12.55)	0.35 (0.09-0.64)	0.56 (0.38-0.77)	0.55 (0.39-0.91)
p	0.753	0.945	0.064	0.623	0.488
Toys					
Painted wood					
Yes (n=33)	17.46 (11.95-37.85)	5.77 (1.85-8.66)	0.23 (0.07-0.63)	0.62 (0.31-0.77)	0.54 (0.31-0.99)
No (n=38)	25.73 (14.54-40.43)	4.90 (2.72-11.72)	0.28 (0.11-0.46)	0.53 (0.32-0.77)	0.55 (0.39-0.76)
P	0.493	0.831	0.876	0.818	0.936
Unpainted wood					
Yes (n=41)	25.57 (15.12-40.49)	5.77 (3.19-8.76)	0.23 (0.08-0.51)	0.62 (0.35-0.79)	0.57 (0.35-0.91)
No (n=30)	24.20 (9.98-39.46)	5.01 (1.35-14.26)	0.29 (0.13-0.52)	0.49 (0.31-0.76)	0.51 (0.39-0.78)
P	0.526	0.609	0.522	0.334	0.572
Plush					
Yes (n=60)	22.12 (13.28-36.10)	5.34 (2.86-11.32)	0.29 (0.09-0.54)	0.56 (0.32-0.80)	0.54 (0.39-0.82)
No (n=11)	40.37 (16.26-54.72)	6.05 (1.38-8.55)	0.21 (0.07-0.40)	0.57 (0.27-0.71)	0.56 (0.36-1.09)
p	0.150	0.836	0.321	0.721	0.727

BH4: Tetrahydrobiopterin, BPA: bisphenol A, BPF: bisphenol F, BAZ: BMI for age z-score, DBP: di-buthyl phthalate, DEHP: di-ethylhexyl phthalate, HPA: hyperphenylalaninemia (who blood phenylalanine levels within 2-6 mg/dL without any treatment), MEHP: mono-ethylhexyl phthalate, PA: phenylalanine, PKU: Phenylketonuria, PVC: polyvinyl chloride
Free diet group includes 71 subjects.

A p-value of less than 0.05 was considered to be statistically significant.
